# Hot Deformation Behavior and Dynamic Softening Mechanism in 7B50 Aluminum Alloy

**DOI:** 10.3390/ma16165590

**Published:** 2023-08-12

**Authors:** Ming Li, Yong Li, Yu Liu, Zhengbing Xiao, Yuanchun Huang

**Affiliations:** 1 School of Mechanical and Electrical Engineering, Central South University, Changsha 410083, China; 2 Research Institute of Light Alloy, Central South University, Changsha 410083, China; 3 State Key Laboratory of High Performance Complex Manufacturing, Central South University, Changsha 410083, China

**Keywords:** 7B50 aluminum alloy, hot processing map, DRX mechanism, microstructure evolution

## Abstract

The hot deformation behavior and dynamic softening mechanism of 7B50 aluminum alloy were studied via isothermal compression experiments in the range of 320–460 °C/0.001–1.0 s^−1^. According to the flow curves obtained from the experiments, the flow behavior of this alloy was analyzed, and the Zener–Hollomon (Z) parameter equation was established. The hot processing maps of this alloy were developed based on the dynamic material model, and the optimal hot working region was determined to be 410–460 °C/0.01–0.001 s^−1^. The electron backscattered diffraction (EBSD) microstructure analysis of the deformed sample shows that the dynamic softening mechanism and microstructure evolution strongly depend on the Z parameter. Meanwhile, a correlation between the dynamic softening mechanism and the lnZ value was established. Dynamic recovery (DRV) was the only softening mechanism during isothermal compression with lnZ ≥ 20. Discontinuous dynamic recrystallization (DDRX) becomes the dominant dynamic recrystallization (DRX) mechanism under deformation conditions of 15 < lnZ < 20. Meanwhile, the size and percentage of DDRXed grains increased with decreasing lnZ values. The geometric dynamic recrystallization (GDRX) mechanism and continuous dynamic recrystallization (CDRX) mechanism coexist under deformation conditions with lnZ ≤ 15.

## 1. Introduction

7B50 aluminum alloy is particularly suitable for the production of high-performance large thick plates and large forgings due to its high strength, high toughness, and excellent hardenability [1,2,3,4,5,6]. With the development of large-scale and integrated structural components in the aerospace field, the 7B50 aluminum alloy with high comprehensive performance will also be further used widely [2,7].

High-temperature plastic deformation is often used to change the geometric shape and optimize the microstructure of 7××× series aluminum alloy materials. The thermal deformation parameters (such as strain, strain rate, and deformation temperature) during high-temperature plastic deformation can significantly affect the flow stress and microstructure evolution of the alloy [8,9,10]. Isothermal compression experiments are often used to investigate the thermal deformation behavior of metals by accurately controlling deformation temperature, strain rate, and deformation amount, and have been used widely in Al-Zn-Mg-Cu aluminum alloys [11,12,13]. Zhao et al. [14] discussed the influence of strain rate on the recrystallization behavior of 7050 aluminum alloy via isothermal compression experiments. The relevant research results indicated that a low strain rate is conducive to the development of DDRX, while CDRX dominates the deformation process of a high strain rate. Zhao et al. [15] developed two different constitutive models based on the influence of strain rate. Furthermore, Zhao et al. [16] investigated the effect of the forging rate on 7050 aluminum alloy bracket forgings. Li et al.’s study [17] on hot compression experiments of Al-Zn-Mg-Cu alloy showed that CDRX under low temperature and high strain rates is mainly composed of micro-shear band assist and progressive lattice rotation near grain boundaries. Xu et al.’s research [18] on Al-Zn-Mg-Cu alloy showed that the increase of Z parameter will lead to an increase in the number of misorientation angles and low angle grain boundaries (LAGBs), while the dislocation density will decrease gradually.

Compared with 7050 aluminum alloy, 7B50 aluminum alloy appropriately increases the content of Zn and Mg elements, and further reduces the content of impurity elements, such as Fe and Si, by improving the melting and casting processes. Related studies [19,20,21] have confirmed that increasing the content of Zn and Mg elements can simultaneously improve the strength of 7××× aluminum alloy. In addition, the decrease in Fe and Si impurity element content can reduce the formation of hard and brittle second phase and improve the plastic formability of 7××× aluminum alloy [22]. 7B50 aluminum alloy for aviation is usually prepared into thick plates or large forgings via hot deformation processing methods such as hot rolling or hot forging. Therefore, the experimental research of the hot deformation behavior of 7B50 aluminum alloy can help to control the microstructure and improve the performance of the product. However, there are currently few reports on the hot deformation behavior of 7B50 aluminum alloy and the influence of Z parameters on microstructure evolution. Therefore, an in-depth understanding of the effects of the deformation parameters on the deformation behavior and microstructure of 7B50 aluminum alloys is required to manufacture 7050 aluminum alloy components with microstructure and properties that meet the requirements.

In order to investigate the hot deformation behavior and microstructure evolution of 7B50 aluminum alloy, isothermal compression experiments were conducted on the 7B50 aluminum alloy using a Gleeble-3800 isothermal simulation machine in the temperature range of 320–460 °C and the strain rate range of 0.001–1.0 s^−1^. In this paper, the influence of deformation parameters on flow behavior is discussed using isothermal compression experiments on 7B50 aluminum alloy. Based on the Arrhenius constitutive model, the thermal deformation activation energy of 7B50 aluminum alloy was obtained, and the Z parameter equation of the alloy was established. The optimum hot processing parameter range of 7B50 aluminum alloy was determined by constructing its hot processing maps. Based on the EBSD microstructure analysis, the influence of Z parameters on microstructure was discussed and the relationship between Z parameters and the dynamic softening mechanism was established. The research work in this paper provides a basis for reasonably formulating the hot working process of 7B50 aluminum alloy and controlling the microstructure and properties of the product.

## 2. Materials and Methods

### 2.1. Experimental Materials and Isothermal Compression Tests

The experimental material was commercial 7B50 aluminum alloy produced by Chinalco Southwest Aluminum Co., Ltd. (Chongqing, China) The specific chemical composition is shown in Table 1.

**Figure 1:**
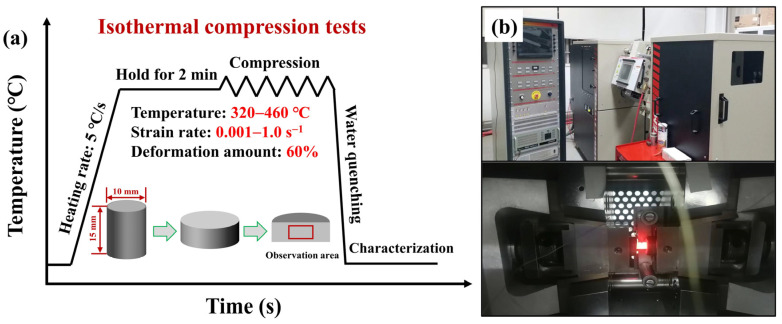
(**a**) Schematic diagram of isothermal compression and (**b**) the Gleeble isothermal simulation machine.

In order to reduce the composition segregation and improve the uniformity of the ingot microstructure, the as-cast 7B50 aluminum alloy was subjected to a two-step homogenization treatment at 400 °C/10 h + 470 °C/48 h. Hot compression standard specimens with a dimension of ϕ10 × 15 mm were machined via wire cutting. The Gleeble-3800 isothermal simulation machine (as shown in Figure 1b) was used for isothermal compression experiments, and the schematic diagram of isothermal compression is depicted in Figure 1. The temperature range was 320–460 °C, the strain rate range was 0.001–1.0 s^−1^, and the compression deformation of the sample was 60% for the isothermal compression experiments. Graphite sheets were placed between the two end faces of the experimental sample and the moLd for lubrication. Before isothermal compression, the experimental sample was heated to the required temperature at a rate of 5 °C/s and held for 2 min to eliminate the temperature gradient. Water cooling was carried out immediately after compression to retain the deformed microstructure.

### 2.2. Microstructure Characterization

The sample after isothermal compression was symmetrically split along the compression direction (CD), and the microstructure at the center of the sample thickness position was characterized. The specimens used for observation were sequentially ground with sandpaper to 2000 grit and then mechanically polished. After mechanical polishing, the samples for EBSD observation were subjected to electropolishing treatment on a double-jet thinning instrument. The electropolishing solution was a mixture of 70% alcohol and 30% nitric acid. The temperature and voltage of electropolishing were −25 °C and 15V, respectively. The scanning electron microscope used for microstructure detection was the TESCAN MIRA3 equipped with an electron backscatter diffraction (EBSD) system. EBSD data was processed via channel5 software.

## 3. Results and Discussion

### 3.1. Initial Microstructures

**Figure 2:**
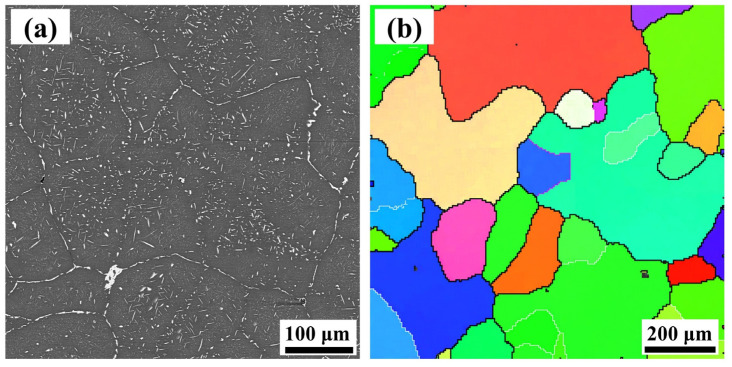
The initial microstructures of as-cast 7B50 alloy: (**a**) SEM image, (**b**) EBSD map.

The SEM and EBSD images of 7B50 aluminum alloy before hot deformation are displayed in Figure 2. The eutectic structure of as-cast 7B50 aluminum alloy partially dissolves during the two-step homogenization heat treatment (400 °C/10 h + 470 °C/48 h). Subsequently, a large number of fine Mg(Al, Cu, Zn)_2_ phases precipitated inside the grains during the slow cooling process. Based on the inverse pole figure (IPF) diagram obtained by the EBSD system (Figure 2b), the microstructure before deformation is composed of coarsely equiaxed grains. The average grain size of as-homogeneous 7B50 aluminum alloy was calculated to be ~174 μm using the line intercept method.

### 3.2. Flow Behavior

#### 3.2.1. Flow Stress Curves

**Figure 3:**
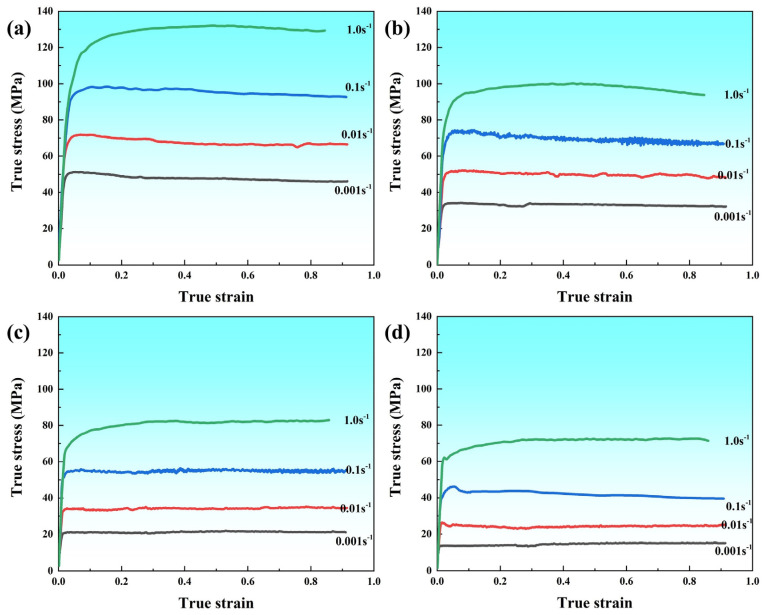
True stress-strain curves under different deformation conditions during isothermal compression: (**a**) 320 °C, (**b**) 370 °C, (**c**) 420 °C, (**d**) 460 °C.

Figure 3 displays the flow stress curves of 7B50 aluminum alloy during hot compression at 320–460 °C/0.001–1.0 s^−1^. In the early stage of deformation, all flow curves rise rapidly, which is a typical work-hardening phenomenon caused by the proliferation of dislocation density [23]. However, the subsequent flow behavior is highly sensitive to the strain rate and deformation temperature. At low temperatures and high strain rates, the flow curves increase slowly with deformation, and there is no peak phenomenon in the flow stress curves. At high temperatures and low strain rates, the flow curves quickly reach their peaks and then slowly decrease and stabilize. The differences in flow stress curves are closely related to the dynamic softening mechanisms (DRV and DRX) during deformation [23,24]. As is well known, the decrease in dislocation density during hot deformation is caused by the dynamic softening behavior of the alloy. The different softening mechanisms mediated by the deformation temperature and strain rate differ in the rate and method of eliminating dislocation density, ultimately leading to significant differences in the flow stress curves.

#### 3.2.2. The Zener–Hollomon Parameter

**Figure 4:**
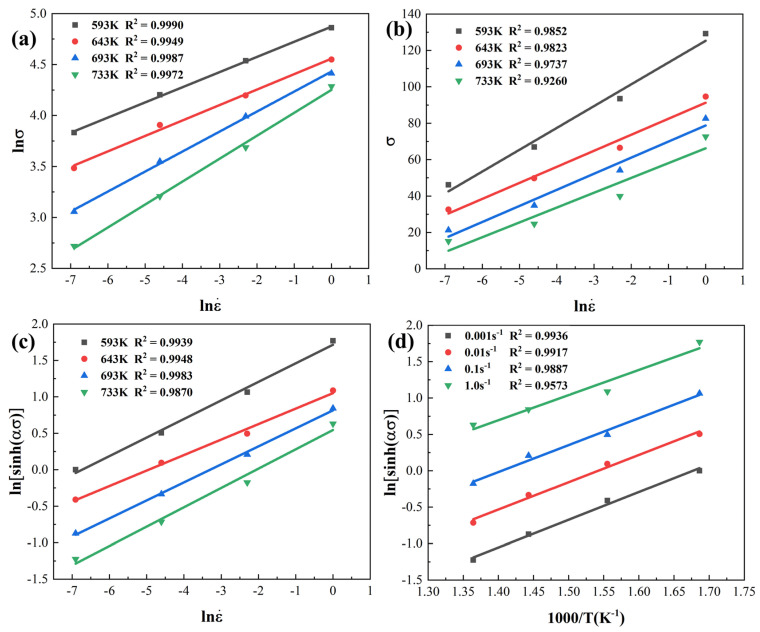
Linear fitting of the relationship between (**a**) $ln\dot{\varepsilon }$-lnσ, (**b**) $ln\dot{\varepsilon }$-σ, (**c**) $ln\dot{\varepsilon }$-$ln\left[sinh\left(\alpha \sigma \right)\right]$, and (**d**) $ln\left[sinh\left(\alpha \sigma \right)\right]$ − 1/T under different deformation conditions.

Zener and Hollomon jointly proposed using an exponential equation to describe the comprehensive effect of strain rate and deformation temperature and the equation of Z parameter is as follows [18]:

(1) $$Z=\dot{\varepsilon }exp\left(\frac{Q}{RT}\right)$$

where $\dot{\varepsilon }$ (s^−1^) and T (K) are the strain rate and deformation temperature, R represents the gas constant (8.314 kJ/moL), and Q (kJ/moL) represents the activation energy of deformation, which describes the difficulty of deformation. Furthermore, the Arrhenius constitutive model commonly used to describe the flow curves can be represented as follows:

(2) $$\dot{\varepsilon }=AF\left(\sigma \right)\exp\left(-\frac{Q}{RT}\right)$$

(3) $$F\left(\sigma \right)=\left\{\begin{array}{cc} \sigma^{N} & \left(\alpha \sigma <0.8\right)\\ exp\left(\beta \sigma \right) & \left(\alpha \sigma \geq 0.8\right)\\ {\left[sin\left(\alpha \sigma \right)\right]}^{n} & \left(For\;all\right) \end{array}\right.$$

where N, β, α (α=β/N), A, and n are material constants. According to Equation (3), the values of N and β are the slope values of the *ln*$\dot{\varepsilon }$-*lnσ*; and *ln*$\dot{\varepsilon }$-*σ*, which can be obtained by linearly fitting the data of Figure 4a,b when the strain is 0.8.

According to Equation (3), the deformation activation energy Q can be expressed as:

(4) $$Q=R{\left[\frac{\partial ln\dot{\varepsilon }}{\partial ln\left[sinh\left(\alpha \sigma \right)\right]}\right]}_{T}{\left[\frac{\partial ln\left[sinh\left(\alpha \sigma \right)\right]}{\partial \left(1/T\right)}\right]}_{\dot{\varepsilon }}=Rns$$

**Figure 5:**
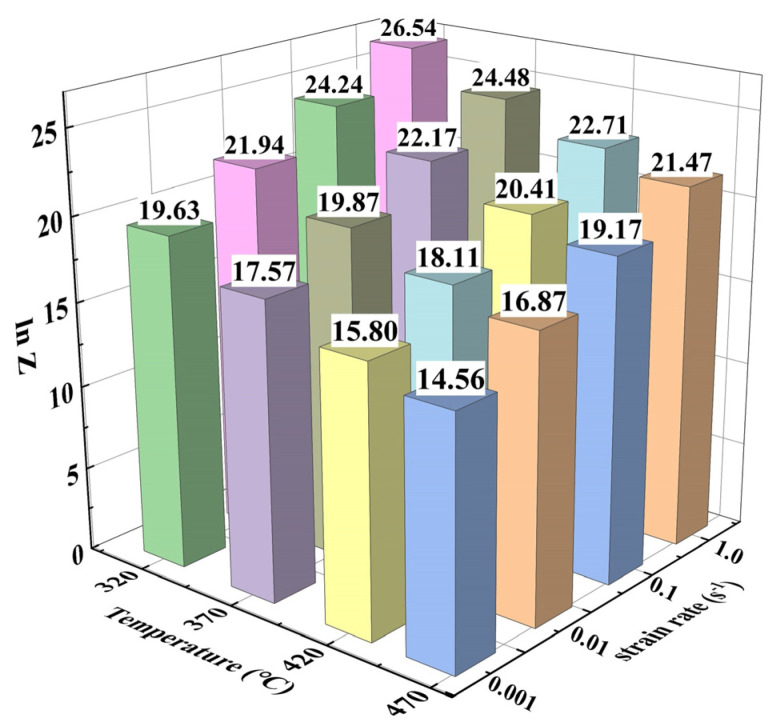
LnZ values under different deformation conditions.

Therefore, based on the obtained α value, the slope values of *ln*$\dot{\varepsilon }$-*ln*[*sinh*(*ασ*)] and *ln*[*sinh*(*ασ*)] − 1/*T* are obtained by linear fitting. The calculated average thermal activation energy Q using the same method is only 130.85 kJ/moL. Numerous scholars have studied the heat deformation behavior of 7050 aluminum alloy (the alloy composition is basically the same as that of 7B50 aluminum alloy). Deng et al. [25] calculated that the Q value of 7050 aluminum alloy after two-step homogeneous heat treatment was 160.3 kJ/moL. The Q value of the solid solution 7050 aluminum alloy after extrusion was confirmed to be 179.53 kJ/moL by Zhao et al. [14]. In addition, Wang et al. [26] investigated the thermal deformation behavior of the Al-6.32Zn-2.10Mg-0.1Cu alloy and showed that the Q value of the alloy after reducing Cu content was still as high as 147.81 kJ/moL. The above data indicate that the 7B50 aluminum alloy in this study has better processing performance compared to the traditional 7050 aluminum alloy. Based on the calculated Q value, the lnZ values under different conditions can be obtained using Equation (1) and are shown in Figure 5. There is a close correlation between the Z parameter and the dynamic softening mechanism and microstructure. Xu et al. [18] proposed that the Geometric DRX dominated at lnZ ≤ 23.44, CDRX dominated at 23.4 < lnZ < 33.33, and DDRX dominated at lnZ ≥ 33.33 for Al-Zn-Mg-Cu alloy. The results of thermal deformation studies on 7050 aluminum alloy conducted by Zhao et al. [15] showed that the DDRX mechanism gradually transformed into the CDRX mechanism with increasing Z parameter. Next, the effects of Z parameter on the hot working behavior, microstructure evolution, and dynamic softening mechanism were investigated by focusing on hot processing maps and microstructure analysis.

#### 3.2.3. Hot Processing Map

**Figure 6:**
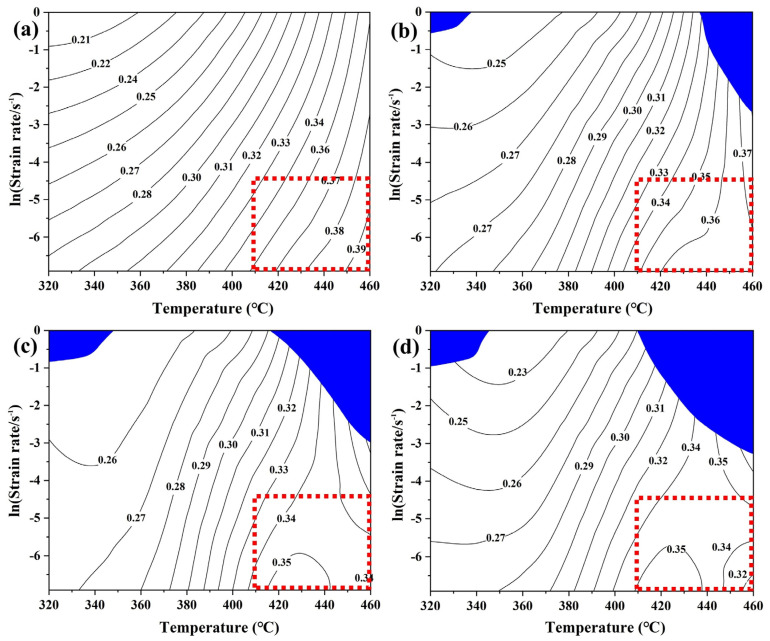
Hot processing maps of 7B50 alloy under different strains: (**a**) 0.2, (**b**) 0.4, (**c**) 0.6, (**d**) 0.8.

Hot processing maps based on the dynamic material model are widely used to guide the hot plastic working of aluminum alloys [26,27]. Figure 6 illustrates the hot processing maps under different strain conditions, and the details of establishing the hot processing maps can be referred to in the literature [18,28,29,30,31]. In the figure, the blue area represents the unstable processing domains that should be avoided during hot plastic processing. The blue unstable domains are mainly distributed in the high strain rate range and increase progressively with increasing strain. The contour lines in the figure are the power dissipation efficiency (η), which represents the percentage of energy used for microstructure evolution. The optimum processing conditions for the experimental 7B50 aluminum alloy can be seen in the hot processing maps as 410–460 °C/0.01–0.001 s^−1^.

It is well known that aluminum alloys belong to high stacking fault energy metals, which are prone to dislocation climb and cross-slip due to the narrow expansion width of dislocations. Therefore, DRV, rather than DRX, usually dominates the thermal deformation process of aluminum alloys. However, the significant dynamic recrystallization behavior of 7××× aluminum alloy has also been reported in much of the literature due to the addition of large numbers of various alloying elements [32,33,34,35,36]. Ren et al.’s research [37] shows that the dynamic softening mechanism of 7055 aluminum alloy gradually changes from DRV to DRX as the η value increases. When the η value is greater than 50%, the microstructure is composed of fully dynamic recrystallized grains. The range of power dissipation efficiency values in this study is 0.22–0.36, which may lead to the occurrence of DRV and partial DRX.

### 3.3. Microstructural Evolution and Dynamic Softening Behavior

#### 3.3.1. At High lnZ Values (lnZ ≥ 20)

**Figure 7:**
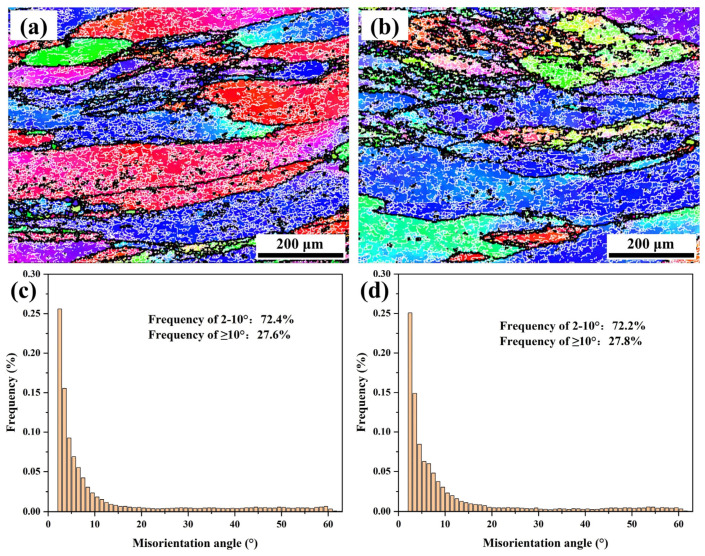
IPF maps and misorientation angle distributions at high lnZ values: (**a**,**c**) lnZ = 22.71 (420 °C, 1 s^−1^), (**b**,**d**) lnZ = 20.41 (420 °C, 0.1 s^−1^).

Figure 7 shows the IPF maps and grain boundary misorientation angle distribution diagrams under the deformation conditions of 420 °C/1.0 s^−1^ and 420 °C/0.1 s^−1^ (lnZ ≥ 20). In the IPF maps, the high-angle grain boundaries (HAGBs) with a misorientation angle greater than 10° is represented by a black solid line, while the white solid line is the low-angle grain boundaries (LAGBs) with a misorientation angle of 2–10°. As can be seen, the microstructure under high lnZ values exhibits typical deformation characteristics. The coarse original grains are elongated perpendicular to the compression direction. There are a large number of distributions of discontinuous and staggered LAGBs within the matrix, which have not formed obvious sub-grains. The grain boundary misorientation angle distributions in Figure 7c,d show that the proportion of LAGBs is as high as 72.4% and 72.2%, which is much higher than the proportion of LAGBs before deformation (18.2%). Meanwhile, the uniform distribution of LAGBs within the matrix indicates that the experimental alloy undergoes uniform deformation during isothermal compression.

**Figure 8:**
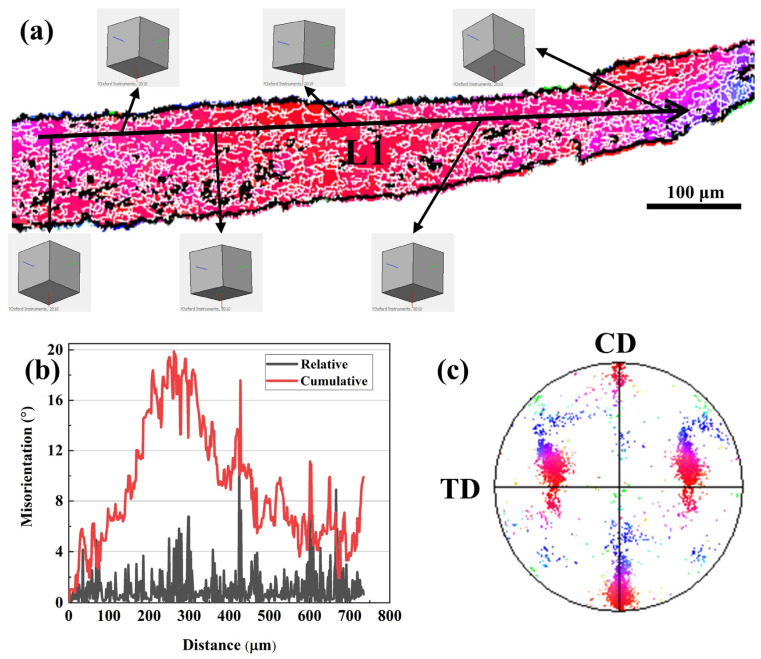
Deformation behavior of individual grain at lnZ = 22.71 (420 °C, 1 s^−1^): (**a**) IPF map and crystal orientation model, (**b**) relative and cumulative misorientation angle, and (**c**) {001} PF.

The deformation behavior of individual grains under the deformation condition of lnZ = 22.71 (420 °C, 1 s^−1^) is presented in Figure 8. The IPF map and crystal orientation models in Figure 8a show that there are orientation differences within the deformed grains. The continuous fluctuation of the relative misorientation angle along arrow L1 in Figure 8b indicates that there are a large number of sub-structures with different orientations within the deformed grains. The corresponding cumulative misorientation angle is close to 20°, manifesting that this grain accommodates large plastic deformation [38]. The {001} pole figure (PF) in Figure 8c shows that the {011} < 100 > direction of this grain is parallel to the CD. Dislocation slip causes the distribution of the {001} PF of this grain to shift along the CD after plastic deformation. Therefore, 7B50 aluminum alloy mainly undergoes dynamic recovery softening under the condition of lnZ ≥ 20.

#### 3.3.2. At Middle lnZ Values (15 < lnZ < 20)

**Figure 9:**
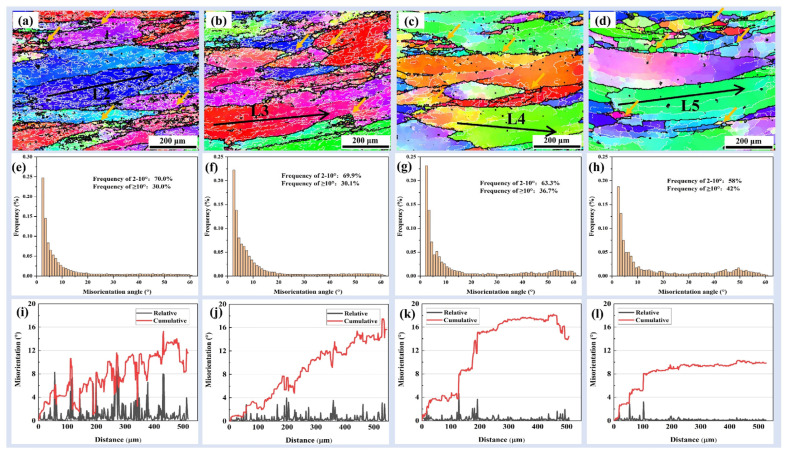
IPF maps, misorientation angle distributions and relative and cumulative misorientation angle at middle lnZ values: (**a**,**e**,**i**) lnZ = 19.63 (320 °C, 0.001 s^−1^); (**b**,**f**,**j**) lnZ = 18.11 (420 °C, 0.01 s^−1^); (**c**,**g**,**k**) lnZ = 17.57 (370 °C, 0.001 s^−1^); (**d**,**h**,**l**) lnZ = 15.80 (420 °C, 0.001 s^−1^).

Figure 9 shows the IPF maps, misorientation angle distributions, and relative and cumulative misorientation angle of lnZ between 15 and 20. Careful observation of Figure 9a–d shows that there are fine DRXed grains at the initial grain boundary (as shown by the yellow arrows). The formation of these DRXed grains is related to the original grain boundary bulging, which is a typical DDRX [39]. The number and size of fine DRXed grains at the initial grain boundaries gradually increase with the decrease in lnZ value. In Figure 9e–h, the proportion of HAGBs increases from 30.0% to 42% as the lnZ value decreases from 19.63 to 15.8. Therefore, 7B50 aluminum alloy mainly exhibits discontinuous dynamic recrystallization under conditions of 15 < lnZ < 20. Another significant feature is that the number of LAGBs inside the grains decreases rapidly with the decreasing in lnZ value, and the distribution of LAGBs gradually changes from discontinuous to continuous. The relative and cumulative misorientation angles of the straight lines L2–L5 within the grains under different lnZ values are shown in Figure 9i–l. The cumulative misorientation angles all exceed 10°, but the fluctuation of relative misorientation angles gradually weakens with the decrease in lnZ value. This indicates that dynamic recovery softening still exists under middle lnZ values.

#### 3.3.3. At Low lnZ Values (lnZ ≤ 15)

**Figure 10:**
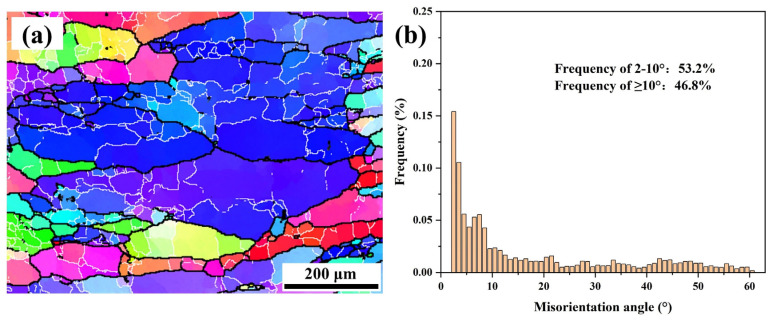
IPF map (**a**) and misorientation angle distributions (**b**) at low lnZ values (lnZ = 14.56, 460 °C/0.001 s^−1^).

Figure 10 shows the IPF map and misorientation angle distribution diagram at lnZ ≤ 15. The microstructure after deformation at 460 °C/0.001 s^−1^ is composed of partial dynamic recrystallization as shown in Figure 10a. The proportion of DRX and the size of DRXed grains are higher than the deformation condition of 15 < lnZ < 20. The proportion of corresponding HAGBs increases to 46.8%. Meanwhile, the LAGBs inside the grains become flat and continuous, and the corresponding proportion of the LAGBs decreases to 53.2%. In addition, a large number of sub-grains composed of LAGBs were formed inside the initial grains. These phenomena indicate that there is a remarkable difference between the DRX mechanism under the condition of lnZ ≤ 15 and 15 < lnZ < 20.

**Figure 11:**
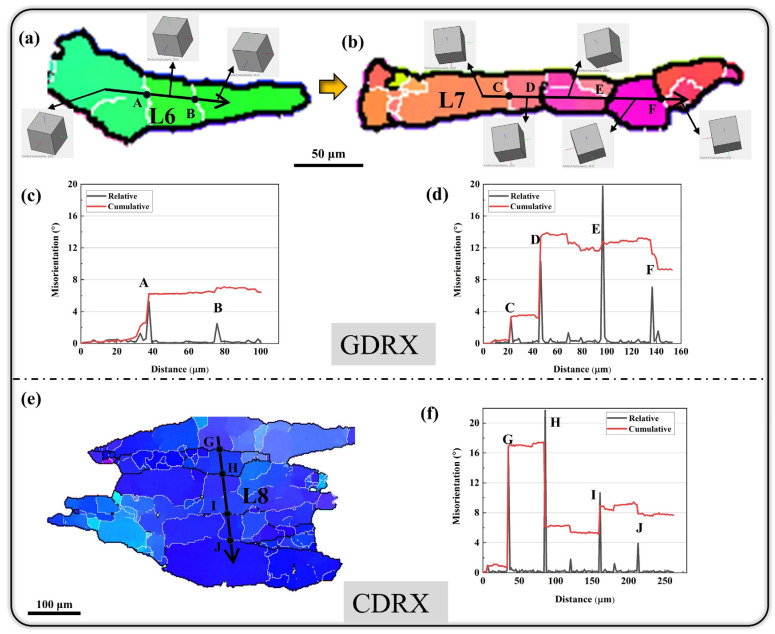
GDRX (**a**–**d**) and CDRX (**e**,**f**) mechanisms at low lnZ values (lnZ = 14.56, 460 °C/0.001 s^−1^).

To investigate the DRX mechanism under deformation conditions of lnZ ≤ 15, the typical regions in Figure 10a were enlarged and displayed in Figure 11a,b,e. As shown in Figure 11a,b, a large number of elongated grains perpendicular to the compression direction are present in the deformation condition of 460 °C/0.001 s^−1^ (Figure 10a). The HAGBs in the sample before deformation are close to each other and almost parallel under compression. Meanwhile, LAGBs perpendicular to HAGBs are formed under the dynamic recovery effect. The elongated deformed grains are divided into brick-like sub-grains by LAGBs as shown in Figure 11a. The relative and cumulative misorientation angles along L6 indicate that the orientation within the sub-grain is basically consistent under the effect of dynamic recovery, while the misorientation angles near the sub-grain boundary are 5.3° (point A) and 2.5° (point B), respectively. In the subsequent deformation process, LAGBs gradually transform into HAGBs as shown in Figure 11b by absorbing the deformation dislocations and sub-grain rotation. The relative misorientation angles along L7 show that the misorientation angles of HAGBs perpendicular to the original grain boundary are 10–20°, which indicate that these HAGBs are transformed by LAGBs [23,27]. These characteristics are consistent with the typical GDRX phenomenon [40,41,42,43]. Therefore, GDRX is the main dynamic softening mechanism under the condition of lnZ ≤ 15.

Furthermore, some CDRX phenomena also exist under the deformation condition of lnZ ≤ 15 as shown in Figure 11e. The CDRX mechanism involves the formation and rotation of sub-grains, which is clearly different from the grain boundary bulging behavior in DDRX mechanism. A large number of sub-grains consisting of LAGBs are present in Figure 11e. There is a small misorientation between sub-grains as shown in the crystal orientation model. During the subsequent deformation process, the sub-grains are transformed into DRXed grain via rotation, and the low-angle sub-grain boundaries are also transformed into HAGBs (as shown in Figure 11f). Therefore, both GDRX and CDRX mechanisms exist under low lnZ values.

## 4. Conclusion

In this paper, the hot deformation behavior and dynamic softening mechanism of 7B50 aluminum alloy were systematically studied via isothermal compression experiments at 320–460 °C/0.001–1.0 s^−1^. The main research findings are as follows:

1. Based on the Arrhenius conservative equation, the deformation activation energy of 7B50 aluminum alloy was calculated to be 130.85 kJ/moL. Meanwhile, the Z parameter equation of this alloy was established.
2. Based on the dynamic material model, the hot processing maps of 7B50 aluminum alloy were established. The optimal processing parameter range for 7B50 aluminum alloy was determined to be 410–460 °C/0.01–0.001 s^−1^.
3. The DRV softening phenomenon exists in all deformation conditions of 7B50 aluminum alloy. However, DRV is the only softening mechanism at lnZ ≥ 20. DDRX is the dominant dynamic recrystallization mechanism under deformation conditions of 15 < lnZ < 20. The size and proportion of DDRXed grains increased with decreasing lnZ values. Both GDRX and CDRX mechanisms exist under deformation conditions of lnZ ≤ 15.

## Figures and Tables

**Table 1:** Chemical compositions of 7B50 aluminum alloy (wt. %).

Zn	Mg	Cu	Zr	Ti	Fe	Si	Al
6.35	2.15	2.10	0.10	0.02	0.06	0.03	Bal.

## Data Availability

The raw/processed data required to reproduce these findings cannot be shared at this time due to legal or ethical reasons.
